# Impact of Dark Triad on Anxiety Disorder: Parallel Mediation Analysis During Pandemic

**DOI:** 10.3389/fpsyg.2022.914328

**Published:** 2022-07-04

**Authors:** Liu Shengbo, Muhammad Fiaz, Yasir H. Mughal, Worakamol Wisetsri, Irfan Ullah, Diandian Ren, Alina Kiran, Kavindra Kumar Kesari

**Affiliations:** ^1^School of Higher Education, Dalian University of Technology, Dalian, China; ^2^Department of Management Science, Qurtuba University of Science and Information Technology, Dera Ismail Khan, Pakistan; ^3^Department of Health Administration, College of Public Health and Health Informatics, Qassim University, Buraydah, Saudi Arabia; ^4^Department of Manufacturing and Service Industry Management, Faculty of Business and Industrial Development, King Mongkut's University of Technology North Bangkok (KMUTNB), Bangkok, Thailand; ^5^Beijing Institute of Technology, Beijing, China; ^6^School of Banking and Finance, University of International Business and Economics, Beijing, China; ^7^Department of Technology and Management, Universiti Teknikal Malaysia Melaka, Malacca, Malaysia; ^8^Department of Applied Physics, School of Sciences, Aalto University, Espoo, Finland; ^9^Department of Bio Physics and Bio Systems, School of Chemical Engineering, Aalto University, Espoo, Finland

**Keywords:** narcissism, Machiavellianism, psychopathy, ASMR, loneliness, anxiety disorder

## Abstract

The current study aimed to investigate the mediating role of loneliness, ASMR, on the relationship between narcissism, Machiavellianism, and psychopathy and anxiety disorder. The population of the study was professionals working in public and private sector organizations. Non-probability snowball technique was used. Data was collected from 512 professionals. A total of 653 questionnaires were distributed and 512 were used in the analysis yielding a response rate of 78.4%. PLS-SEM was used to analyze the data. Measurement and structural models were developed to test the reliability, and validity of the scales as well as hypotheses. Findings of the measurement model revealed that scales were reliable and valid while results of the structural model revealed that narcissism, psychopathy, COVID loneliness, and ASMR have a significant direct impact on anxiety disorder but Machiavellianism does not have a significant effect on anxiety disorder. In addition, COVID loneliness and ASMR mediated between narcissism and psychopathy but do not significantly mediate between Machiavellianism and anxiety disorder. The current study has extended the body of knowledge by bridging the two theories theory of attachment and cognitive dissonance theory. The current study has provided the primary evidence that COVID loneliness increases anxiety while ASMR (audio-visual) tingling sensations help to reduce anxiety disorder.

## Introduction

The COVID-19 pandemic affected everyone, especially those in the realm of service sectors. Lockdown and the COVID pandemic impacted social and mental health (Babore et al., 2020; Sood et al., 2020). The state's lockdown created social isolation, quarantine, and separation from loved ones, leading to feelings of loneliness. That finally leads to anxiety disorder and mental health issues. Certain personality disorders enhance the likelihood of loneliness, anxiety, and depression (Hardin et al., 2021), and the most severe of these is the dark triad (Kowalski, 2001). Recently, it has been observed that individuals with the dark triad have had issues obeying government regulations (Nowak et al., 2020; Zajenkowski et al., 2020; You, 2021). While research showed that for battling sensations of being lonely and random dark moods individuals are increasingly employing autonomous sensory meridian response (ASMR) stuff to relax and alleviate anxiety and depression. A recent study demonstrates that ASMR-capable persons have high trait neuroticism, which is associated with unpleasant feelings like anxiety (Eid et al., 2022). Relatively little attention has been paid to individuals with anxiety disorder specifically those who possessed dark characteristics suffering from social loneliness due to deadly COVID 19. This is particularly surprising since individuals with anxiety disorder experience intense feelings of loneliness. But the question remains unanswered how to cope with distinct intrinsic evils i.e., being a dark personality, social loneliness, and severe situation of anxiety disorder triggered due to a deadly virus? So, by bridging two theories attachment theory (Ainsworth and Bowlby, 1991), and the theory of cognitive dissonance (Festinger, 1957), the present study attempts to compare the Dark Triad of personality characteristics between persons who experienced pandemic limitations and to examine the association of its component qualities with the intensity of anxiety symptoms. It allows us to examine whether loneliness and ASMR triggers affect/mediated the vulnerable anxiety disorder in each of the dark personality traits? Given that previously none of the studies examined the subject matter stressed above. So, it is essential to investigate whether loneliness and ASMR during the COVID-19 pandemic influenced low or high those who possessed dark personality disorder and suffered from an anxiety disorder. So, the current study offers a novel contribution by extending the body of knowledge and considering the probable correlations between ASMR and loneliness, personality characteristics, and behavioral wellness should help improve COVID-related evaluations.

The contribution of the current study lies in bridging two theories the attachment theory and second theory of cognitive dissonance. Ainsworth and Bowlby (1991) proposed the attachment theory. In stressful situations, attachment theory describes a person's attitude toward others (Bretherton and Munholland, 1999). Detachment can increase an individual's survival skills and capacities (Bretherton and Munholland, 1999). This theory responds to environmental threats by promoting caregiver proximity (Nolte et al., 2011). Losing an attachment figure can aggravate loneliness, depression, and anxiety (Sbarra and Hazan, 2008). Connecting with attachment figures or recalling earlier coping skills might help alleviate anxiety (Diamond and Aspinwall, 2003; Hill, 2009).

The cognitive dissonance theory was proposed by Festinger (1957). The notion was founded on human mentality and inherent psychological inconsistencies. For a person with mental instability, has only two choices: strive or die (Festinger and Carlsmith, 1959). Long-term mental irregularity may alleviate stress and anxiety (Festinger, 1957). Cognitive dissonance is a condition of mental tension and discomfort (Harmon-Jones and Mills, 2019). Which ultimately generates emotions, beliefs, actions, values, and environment (Cooper, 2007). When a person's beliefs collide with change, motivation emerges to address the conflict (Thibodeau and Aronson, 1992). These theories are helpful to explain how social distance such as loneliness can increase anxiety and how to overcome this anxiety through ASMR among narcissists, Machiavellians, and psychopaths (Fang et al., 2021).

The dark triad (narcissism, Machiavellianism, and psychopathy) are three socially aversive personality traits that lead to manipulative attitudes, self-serving motives, selfishness, callousness, and unethical, or immoral behavior like a fraud (Jones and Paulhus, 2014). Narcissism is characterized by arrogance, self-absorption, lack of empathy, and superiority (Morf and Rhodewalt, 2001). Narcissists like being admired or judged by their appearance (Wallace et al., 2002). In severe situations, such as the COVID-19 pandemic (Gómez-Leal et al., 2019), narcissists were more confident. Even when lonely or in danger, narcissists reported less anxiety (Watson and Biderman, 1993). However, grandiose narcissism has been found to generate unhappiness and distress (Gómez-Leal et al., 2019; Fang et al., 2021). According to narcissists want social approval to survive, yet they are irritated by rage and discontent with their surroundings. Because of their dysfunction, they are unable to perform in social, professional, or romantic situations (Vaknin, 2020). Machiavellianism defined unpleasant conduct as increased political behavior, insincerity, callousness, frosty look, and lack of ethics (Christie and Geis, 1970). Those with more Machiavellian characteristics used deceit to advance (Jonason et al., 2015; Gómez-Leal et al., 2019). However, Sabouri et al. (2016) argued that comparatively females with higher Machiavellian traits were found to be more depressed and reported greater anxiety symptoms than non-depressed Machiavellians, while Zhang et al. (2015) observed that social loneliness is a key predictor of anxiety and depression in those who possessed dark personality traits. Similarly, Bakir et al. (1996) found a significant association between anxiety and Machiavellian only in male samples. While, Skinner (1982) showed that both male and female Machiavellians exhibited higher degrees of depression and anxiety than the general population with moderate personality traits (Ali and Chamorro-Premuzic, 2010). Psychopathic traits lack empathy, anxiety, sadness, and regret (Hare, 1985). According to Derefinko (2015), some psychopaths have a higher level of anxiety symptoms while Durand and Plata (2017) argued that psychopathy is often associated with fearlessness and blunted emotions. Interestingly, Skeem et al. (2007) evaluated seventeen studies on psychopathy and anxiety disorders and found evidence of causing fear/anxiety in the core of psychopathy. However, Dutton (2012) explored psychopaths' both positive and negative personality characteristics. Psychopathic personalities susceptible to police enforcement, business, politics, and the military have great self-esteem, can handle pressure, and tolerate anxiety (Lykken and Tellegen, 1996). To sum up, it can be concluded that psychopathy seems to be a neuropsychiatric disorder marked by a lack of emotional responses, empathy, and control over behavior, which often leads to anxiety and depression.

Loneliness is a rising human emotion causes anxiety and depression (Chatterjee, 2018). Over half of Americans say they are lonely every day, yet just 53% report considerable in-person engagement (Cacioppo et al., 2015). Loneliness is a social void (Perlman and Peplau, 1984). Social ties affect mental health (Hawkley et al., 2003; Masi and Brovedani, 2011). Many people, even when surrounded by loved ones, need genuine social interaction (Cacioppo et al., 2015). Loneliness is associated with suicidal ideation, sadness, anxiety, and obesity (Cacioppo et al., 2015). Social anxiety disorder (SAD) patients fear negative evaluations. The poor self-esteem and avoidance behaviors of SAD sufferers impede meaningful social relationships (Alden and Taylor, 2004; Piccirillo et al., 2016; Hoffman et al., 2021). Anxious social people are lonely (Teo et al., 2013; Cacioppo et al., 2015). However, Meltzer (2013) claim that loneliness causes all dissociative disorders. Anxiety condition was the only predictor of future loneliness in longitudinal research (Lim et al., 2016). Moreover, Cecchetto et al. (2021) examined participants' physical, psychological, emotional, and social (income, workload conditions) and found greater emotional eating and binging. Emotional and binge eating was associated with higher body mass index (BMI) and alexithymia. Pandemic phase 2 saw fewer binges and emotional eating. Weakened mental health and eating habits tend to be linked.

ASMR is a pleasant, head-focused tingling sensation that happens in response to audiovisual stimuli, causing feelings of relaxation and pleasure (Barratt and Davis, 2015; Roberts et al., 2021). ASMR triggers include whispering, harsh sounds, and tapping (Barratt and Davis, 2015; Poerio et al., 2018). Contrary to Roberts et al. (2019) findings, sensory-affective sensitivity (SPS) still accounts for certain ASMR stimulus choices. High empathy and heightened attention to things may explain why people prefer nurturing roleplay stimuli. A higher level of openness and absorption in ASMR experiences than in controls suggests that ASMR signals a predisposition toward SPS. According to Barratt and Davis (2015), 98% of ASMR users say it helps them relax, 82% say it helps them sleep, and 70% say it helps them deal with anxiety. ASMR has been demonstrated to help relieve feelings of anxiety and stress that interfere with daily tasks (Kovacevich and Huron, 2019). Furthermore, Roberts et al. (2021) investigated ASMR's relationship to major personality factors. However, there were no changes in mindfulness or flow between ASMR experiencers and age- and gender-matched controls. Park and Lim (2021) studied ASMR's effect on college students' anxiety, sleep, and stress. In the ASMR group, there was less discomfort. The ASMR treatments reduced anxiety in the test group. While Harrison (2017) also investigates ASMR-like audiovisual material for depression and anxiety. An ASMR and an existing audiovisual entrainment technique were tried in video meditation. Mood and relaxation were tested before and after the video. Mentally depressed persons and the general public both improved.

Humans are a social species that need socialization and cooperation to thrive (Young et al., 2008). Loneliness and social collapse produce resentment (Berkowitz, 1989). Loneliness can change a person's dark side of personality (Masui, 2019). Social isolation due to loneliness may cause psychopathic people to act more aggressively than non-psychopathic people (Zhang et al., 2015; Masui, 2019). Psychopathic and Machiavellian personalities had higher loneliness tendencies than narcissistic types (Zhang et al., 2015). This disparity may be related to grandiose sentiments (Petrides et al., 2011). Moreover, Zhang et al. (2015) revealed that only loneliness was fully mediated by Machiavellianism and emotional intelligence, whereas narcissism and loneliness had lesser mediation. According to Jonason and Krause (2013), Machiavellian types might suffer from loneliness if they avoid social events. Narcissists can easily regulate their dissatisfaction, worry, and anxiety owing to adaptive qualities and self-admiration (Jonason and Krause, 2013). While, Osimo et al. (2021) investigated the impact of personality traits such as alexithymia and resilience on anxiety, stress, and depression during COVID lockdown. Two distinct groups of people had favorable and negative emotional reactions (negative ER). Limitations removed from those with high resilience reduced stress. Anxiety and stress are connected to these qualities. The same study found that boosting a person's capacity to cope or highlighting their alexithymia inclinations and personality features might lower anxiety and depression symptoms.

It is now feasible to characterize ASMR experiences, which are psychologically relaxing triggers that repeat and control anxiety, despair, boredom, and other core emotions (Baker, 2018). ASMR videos include dancing competitions, haircuts, massage tutorials, and customer service role plays, that can ultimately alleviate sorrow, depression, and anxiety (Baker, 2018; Eid et al., 2022). Moreover, Gomes Arrulo et al. (2021) looked at the impact of music listening and dark personality characteristics on stress perception and cognitive abilities. Both classical and hip-hop/rap music can assist reduce stress. But narcissism produces anxiety and disappointment. Classical music may soothe them. Results found that listing music improved narcissists' performance but raised their stress. Depending on musical interests, narcissism may improve stress responses or cognitive function. Similarly, Bowes et al. (2018) explored the relationship between music and movie genre preferences and psychopathic subdimensions. These correlations were compared to other personality disorders or traits. Few psychopathy subdimensions' associations with entertainment choices were accurate enough to pique participants' curiosity. Extroversion and openness to new experiences were slightly associated with entertainment interests, but not entitlement/exploitation and Machiavellianism. So, the question arises when and how individuals with dark triggers can cope with anxiety disorders that arise due to COVID limitations. So, from the above, we posit that:

***H***_**1*a***_***:*** *Narcissism has a positive effect on anxiety disorder*.

***H***_**1*b***_***:*** *Machiavellianism has a positive effect on anxiety disorder*.

***H***_**1*c***_***:*** *Psychopathy has a positive effect on anxiety disorder*.

***H***_**2**_***:*** *Loneliness has a positive effect on anxiety disorder*.

***H***_**3**_***:*** *ASMR has a significant effect on anxiety disorder*.

***H***_**4*a***_***:*** *Narcissism and anxiety disorder are mediated by Loneliness*.

***H***_**4*b***_***:*** *Machiavellianism and anxiety disorder is mediated by Loneliness*.

***H***_**4*c***_***:*** *Psychopathy and anxiety disorder is mediated by Loneliness*.

***H***_**5*a***_***:*** *Narcissism and anxiety disorder is significantly mediated by ASMR*.

***H***_**5*b***_***:*** *Machiavellianism and anxiety disorder is significantly mediated by ASMR*.

**Figure 1:**
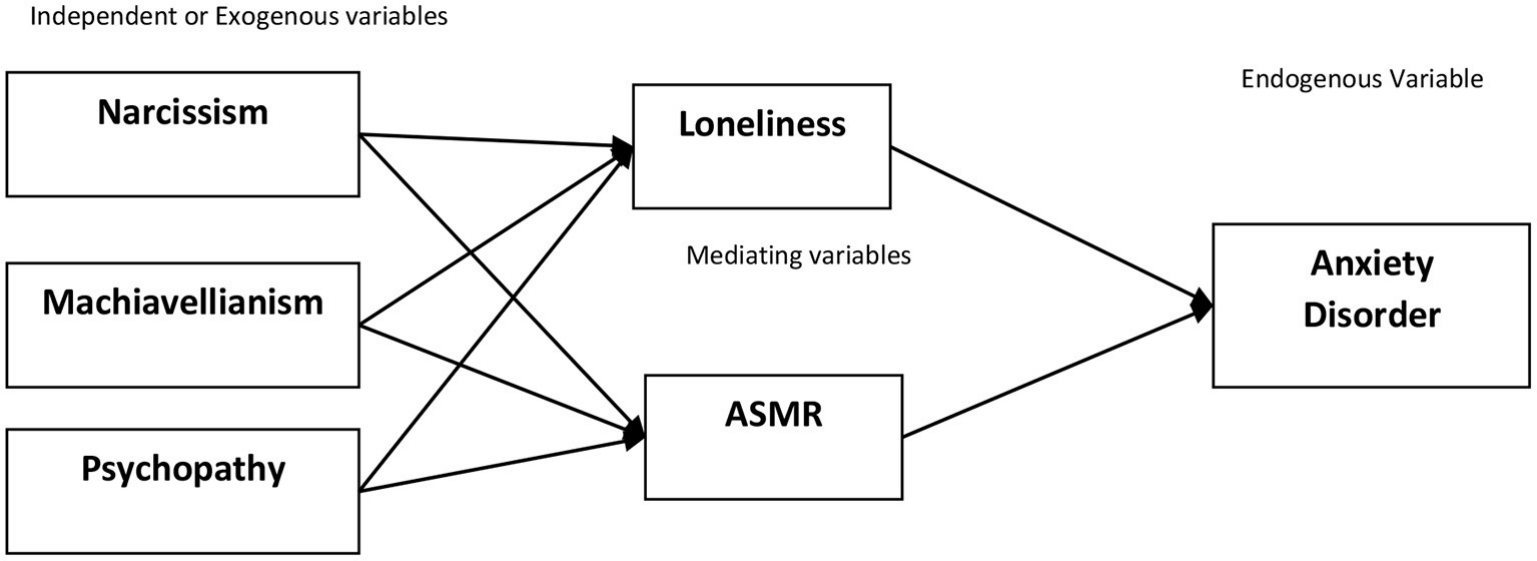
Conceptual framework.

***H***_**5*c***_***:*** *Psychopathy and anxiety disorder is significantly mediated by ASMR* (Figure 1).

## Materials and Methods

### Participants and Sample Size Determination

For this study, data was gathered from a representative sample of Beijing, China's IT, health, and bioinformatics professionals who were affected by COVID-19. A total of 512 participants took part in the study and all of them answered the survey questions. Lockdown and overwork were important contributors to the spread of this deadly virus; thus, we choose to use this strain. Due to the fast spread of the novel coronavirus (COVID-19) pandemic, worldwide death and morbidity rates have surged considerably. As a result of the current global circumstances, an individual's mental health may be endangered. As a result, the goal of this research was to look at the effects of ASMR and COVID loneliness on anxiety symptoms in those who have dark flux.

Sample size determination is always an issue in collecting primary data and survey studies (Kay-Wong, 2013). Hair et al. (2017) has given rules for determining the sample size using PLS-SEM. sample size in PLS-SEM is based on number of arrows from predictors to criterion variables help scholars to calculate sample size. If arrows pointed at latent variable are 2 in that case sample size would be 52, for three arrows sample would be fifty-nine and so on. In the current study number of arrows pointed at criterion variable or latent or dependent variable are 2 so in that study sample size could be 59, in addition Krejcie and Morgan (1970) Table also guide scholars and authors to determine the sample size if population is no known or infinite taking guidance form that table if population is more than one million, then possible size of sample could be 384 but researchers has received 512 responses and used in the analysis. Therefore, it is assumed that enough sample size which represents population of professionals in Beijing China IT market is taken.

### Demographic Information

The majority of the respondents were male 391 (76.4%) followed by female participants 121 (23.6%), further analysis of results revealed that the majority of the respondents were having age of 26-30 years 196 (38.3%) followed by age group of 21–25 years 127 (24.8%) 118 (23%) respondents belong to the age group of 31–35 years and 49 respondents belong to the age of 36–40 years and 22 respondents have an age of more than 40 years respectively. majority of the respondents have experience of up to 3 years i.e. 236 respondents 46.1%, followed by those respondents having experience of 4–6 years 171 (33.4%), 61 respondents (11.9%) have experience of 7–9 years 26 have 10–12 years and 18 have more than 12 years of experience. Table 1 presented demographic information. The majority of the respondents were male 391 (76.4%) followed by private-sector employees 53 (10.4%).

**Table 1:** Demographic information.

**Variables**	**N**	**Percentage**
Male	391	76.4%
Female	121	23.6%
21–25 Years age	127	24.8%
26–30 Years age	196	38.3%
31–35 Years age	118	23%
36–40 Years age	49	9.57%
40 years and above	22	4.29%
3 years' experience	236	46.1%
4–6 Years' experience	171	33.4%
7–9 years' experience	61	11.9%
10–12 years' experience	26	5.07%
12 years and above experience	18	3.51%

### Procedure

With the assistance of COVID-19, we were able to assemble this information from several web sources. Our study's participants were sent a questionnaire via WeChat, a popular messaging app. To inform participants who will be participating in the study about the research objectives, confidentiality regulations, and consent procedures, we used this app. For input, populations of the study were professionals from fields of information technology, health informatics, and bioinformatics professionals from a number of sectors such as teaching in higher education, working in software houses, and research centers, etc. population was so big so non-probability convenience sampling was used to select the sample size. Krejcie and Morgan (1970) table was used to select the sample size. As the Krejcie and Morgan (1970), the table gives a minimum sample size of 384 but researchers have doubled the sample size to have a more accurate picture of the problem thus 768 participants were contacted and 512 completed the survey which are used in the analysis.

We gathered the information in 2 phases. During the first phase, we gathered data on the dark Triad and professional anxiety disorder. The second phase of the study, which took place 4 weeks after the first, collected data on loneliness and ASMR. The researchers then used the database to compile the surveys by cross-referencing the various employee codes. We were able to avert CMB due to this way of data collection (Podsakoff et al., 2003). Phase two of the survey used phone calls to increase the percentage of people who responded (Zhao and Zhang, 2006).

### Instruments

The questionnaire was translated into Chinese and back into English by two bilingual academics, who discussed any issues that arose. A lot of time was spent debating whether or not these measures would be effective in China. There were seven points in the Likert scale from 1 to 7, with 1 representing “strongly disagree” and 7 representing strongly agree, used to score each attribute in the study.

#### Dark Triad

We measured the Dark Triad of personality disorder by the Short Dark Triad measure (SD3) developed by (Jones and Paulhus, 2014). SD3 consists of 27 measuring items having subclinical dimensions of narcissism, Machiavellianism, and psychopathy. the alpha coefficient for the scale was narcissism 0.936, Machiavellianism 0.926, and psychopathy 0.930.

#### Anxiety Disorder

Anxiety disorder was measured through Generalized Anxiety Disorder (GAD7) adopted from Spitzer et al. (2006) consisting of 7 measuring items. The scale's alpha coefficient was 0.903.

#### Loneliness

We measured loneliness by using (Gierveld and Tilburg, 2006) a 6-item brief measure for social loneliness during the COVID pandemic. Alpha for the scale came in at 0.812.

#### ASMR

Data regarding ASMR was collected by using a scale developed by Roberts et al. (2019). The alpha coefficient for the scale was 0.942.

### Data Analysis

In the current study ethical issues were fully complied with. None of the respondents was forced to provide details and the researcher did not indicate their identity. The professionals were therefore guaranteed anonymity and verbal approval was given by the participant.

Partial least square structural equation modeling (PLS-SEM) was used for data analysis (Ringle et al., 2015). There were different options available for model testing i.e., covariance-based software (CB-SEM) that includes Liseral, Mplus, AMOS-SEM, Wrap PLS, or PLS-SEM. As PLS-SEM is more advantageous for users likewise small sample size can be examined through PLS-SEM, and formative and complex mediation models can also be analyzed by PLS-SEM. So, through PLS-SEM all of the structural and measurement models were developed (Hair et al., 2017). As complex mediation modeling was not achievable in SPSS then we used structural equation modeling (SEM). PLS-SEM is the most credible and trustworthy software for complex modeling since it does not assume data normality, sample size, and independence (Min et al., 2020). Factor loading, alpha values, average variance extracted (AVE), and composite reliability (CR) were used to assess scale validity and reliability.

Convergent and discriminatory validity has been observed in the measuring model. Convergent validity was used to assess whether measuring items reported the same idea or not, while discriminant validity was used to examine whether items were different from the construct, or the same (Hair et al., 2014; Ramayah et al., 2018). Discriminant validity was reported by using the hetero-trait and mono-trait (HTMT) ratio. However, according to Black and Babin (2019), the cut-off or threshold level for the (HTMT ratio) is less than 1. The Researchers then proceeded to the structural model to test the study hypotheses (Ramayah et al., 2018).

## Results

PLS-SEM was used for analysis of measurement model. Criteria for measurement were given by Hair et al. (2017). As per recommendations variance inflation factor (VIF) must be less <5, factor loadings of each item should be >0.7, convergent validity i.e., average variance extracted (AVE) must be >0.5 and composite reliability (CR) >0.70 and cronbach alpha must be >0.70. items 4 & 6 of loneliness, items 7 and 15 of ASMR were excluded due to low factor loadings, remaining all items of narcissism, Machiavellianism, psychopathy, loneliness, ASMR, and anxiety disorder met the threshold criteria for VIF, factor loadings, AVE, CR and cronbach alpha given by Hair et al. (2017) thus we assumed that measurement is found reliable and valid (see Table 2).

**Table 2:** Measurement model.

**Items**	**VIF**	**Loadings**	**AVE**	**CR**	**A**
**Narcissism**					
People see me as a natural leader.	2.27	0.73			
I hate being the center of attention.	2.27	0.75			
Many group activities tend to be dull without me.	2.38	0.72	0.62	0.93	0.94
I know that I am special because everyone keeps telling me so.	2.40	0.81			
I like to get acquainted with important people.	2.49	0.83			
I feel embarrassed if someone compliments me.	2.47	0.81			
I have been compared to famous people.	2.56	0.83			
I am an average person.	2.40	0.77			
I insist on getting the respect I deserve.	2.33	0.80			
**Machiavellianism**					
It's not wise to tell your secrets.	2.16	0.76			
I like to use clever manipulation to get my way.	2.15	0.80			
Whatever it takes, you must get the important people on your side.	2.04	0.74			
Avoid direct conflict with others because they may be useful in the future.	2.09	0.77	0.58	0.92	0.93
It's wise to keep track of information that you can use against people later.	2.11	0.75			
You should wait for the right time to get back at people.	2.48	0.75			
There are things you should hide from people to preserve your reputation.	2.25	0.77			
Make sure your plans benefit yourself, not others.	2.60	0.76			
Most people can be manipulated.	2.28	0.78			
**Psychopathy**					
I like to get revenge on authorities.	2.66	0.79			
I avoid dangerous situations.	2.43	0.72			
Payback needs to be quick and nasty.	2.40	0.79			
People often say I'm out of control.	2.27	0.78	0.60	0.93	0.93
It's true that I can be mean to others.	2.29	0.79			
People who mess with me always regret it.	2.24	0.79			
I have never gotten into trouble with the law.	2.17	0.78			
I hate movies where they show blood and guts.	2.20	0.75			
I'll say anything to get what I want.	2.20	0.76			
**Loneliness**					
I experience a general sense of emptiness	1.62	0.71			
There are plenty of people I can rely on when I have problems	1.83	0.75			
There are many people I can trust completely.	1.79	0.71	0.52	0.81	0.81
I miss having people around.	-	-			
There are enough people I feel close to.	1.50	0.71			
I often feel rejected	**-**	-			
**Anxiety disorder**					
Feeling nervous, anxious or on edge	2.08	0.76			
Not being able to stop or control worrying	1.99	0.74			
Worrying too much about different things	2.30	0.75			
Trouble relaxing	2.03	0.72	0.57	0.90	0.90
Being so restless that it is hard to sit still	1.96	0.75			
Becoming easily annoyed or irritable	1.94	0.78			
Feeling afraid as if something awful might happen	2.16	0.78			
**Autonomous sensory meridian response**					
It feels like an altered state of consciousness	1.91	0.77			
It feels like a different state of mind.	2.04	0.72			
It feels as though I have slipped into a hypnotic, trance-like state.	2.24	0.78			
I experience time distortions	2.18	0.72			
I experience an unusual sensation in my head and body.	2.16	0.74			
The sensation feels “tingly”.	2.31	0.74	0.55	0.94	0.94
It feels like Goosebumps on the back of my head.	-	-			
The sensation feels like a “wave of energy”.	2.38	0.75			
The sensation spreads like a wave.	2.14	0.73			
I find the experience calming.	2.39	0.73			
I feel sleepy.	2.34	0.74			
I feel relaxed.	2.30	0.75			
The experience is blissful	2.35	0.77			
I feel euphoric.	-	-			
I find the sensation intensely pleasurable.	2.27	0.73			

*VIF, Variance Inflation Factor; AVE, Average Variance Extracted; CR, Composite Reliability; α = Cronbach Alpha*.

For discriminant validity there are two criteria one is Fornell-Larcker and the second is HTMT ratios. In the current study, HTMT ratios are used for investigating the discriminant validity. Threshold value for HTMT ratios it must be less than 0.85 (Henseler et al., 2015) while as per Hair et al. (2017) it must be less than 1. The above Table 3 shows HTMT ratios met the cut off level given by Hair et al. (2017). Thus, we assume that discriminant validity is established.

**Table 3:** Hetero trait mono trait ratio (discriminant validity).

	**1**	**2**	**3**	**4**	**5**
ASMR					
Anxiety disorder	0.80				
Loneliness	0.82	0.93			
Machiavellianism	0.81	0.86	0.79		
Narcissism	0.81	0.82	0.80	0.94	
Psychopathy	0.79	0.96	0.87	0.92	0.85

To test the hypotheses bootstrapping was run in PLS-SEM. Table 4 presented the results of direct effects. It is evident that Narcissism has a significant and positive influence on anxiety disorder β = 0.30, t = 6.08, *p* < 0.05, LLCI = 0.20, ULCI = 0.39, which means that one unit change in narcissism can bring 30% significant and positive variation in anxiety, furthermore t-statistics is above the threshold and there is no zero between LLCI and ULCI both are positive, therefore, H_1a_ is accepted and substantiated. In addition, Machiavellianism does not influence anxiety disorder, β = 0.01, t = 0.14, *p* > 0.05 LLCI = −0.12, ULCI = 0.15, there is no significant effect of Machiavellianism on anxiety. Hence H_1b_ is rejected. Further psychopathy has a positive and significant impact on anxiety disorder β = 0.42, t = 7.83, *p* < 0.05, LLCI = 0.30, ULCI = 0.52 respectively, it means one percent change in psychopathy could bring 42% possible change in anxiety disorder. Hence H_1c_ is accepted. Loneliness has a significant role on anxiety β = 0.57, t = 9.61, *p* < 0.05 and LLCI = 0.43, ULCI = 0.67, loneliness brings 57% change in anxiety or due to loneliness there are 57% chances that anxiety will be increased. Furthermore, ASMR has significant negative impact on anxiety β = −0.32, t = 5.22, *p* < 0.05 LLCI = 0.21, ULCI=0.45, it means one unit change in ASMR could decrease anxiety upto 32% among respondents. Hence H_2_ & H_3_ are also accepted and substantiated.

**Table 4:** Direct effects.

**Hypotheses**	**B**	**SE**	**t**	**P**	**LLCI**	**ULCI**	**Support**
Narcissism → Anxiety H_1a_	0.30	0.05	6.08	0.00	0.20	0.39	Yes
Machiavellianism → Anxiety H_1b_	0.01	0.07	0.14	0.88	−0.12	0.15	No
Psychopathy → Anxiety H_1c_	0.42	0.05	7.83	0.00	0.30	0.52	Yes
Loneliness → Anxiety H_2_	0.57	0.06	9.61	0.00	0.43	0.67	Yes
ASMR → Anxiety H_3_	−0.32	0.06	5.22	0.00	0.21	0.45	Yes

*β, Beta Value; SE, Standard Error; t, t-statistics>1.96; p, significance value; LLCI, lower limit confidence interval; ULCI, Upper limit Confidence Interval*.

Indirect effects are also investigated through bootstrapping as shown in Table 5. It is revealed from the analysis of results that loneliness mediates between narcissism and anxiety β = 0.17, t = 4.19, *p* < 0.05, LLCI = 0.10, ULCI = 0.27, which means that due to loneliness and narcissism there is a 17% possibility of variation in anxiety disorder. Hence H_4a_ is accepted. Loneliness was used as a mediator between Machiavellianism and anxiety but loneliness does not play a significant role between Machiavellianism and anxiety β = −0.04, t = −0.70, *p* > 0.05, LLCI = −0.14, ULCI = 0.06 there is no significant indirect effect of loneliness between Machiavellianism and anxiety, Hence H_4b_ is rejected. Loneliness mediates between psychopathy and anxiety i.e. β = 0.33, t = 5.82, *p* < 0.05, LLCI = 0.21, ULCI = 0.43, one unit change in loneliness and psychopath could bring 33% change in anxiety. Hence H_4c_ is accepted. Furthermore, ASMR was also used as a mediator between narcissism and anxiety β = 0.13, t = 4.06, *p* < 0.05, LLCI = 0.07, ULCI = 0.20, which is positive and significant it means that listening and watching ASMR videos could decrease anxiety upto13% among respondents, H_5a_ is accepted.

**Table 5:** Indirect effects (mediation results).

**Hypotheses**	**β**	**SE**	**T**	**p**	**LLCI**	**ULCI**	**Support**
Narcissism → Loneliness → Anxiety H_4a_	0.17	0.04	4.19	0.00	0.10	0.27	Yes
Machiavellianism → Loneliness → Anxiety H_4b_	−0.04	0.05	−0.70	0.44	−0.14	0.06	No
Psychopathy → Loneliness → Anxiety H_4c_	0.33	0.06	5.82	0.00	0.21	0.43	Yes
Narcissism → ASMR → Anxiety H_5a_	0.13	0.03	4.06	0.00	0.07	0.20	Yes
Machiavellianism → ASMR → Anxiety H_5b_	0.05	0.03	1.50	0.13	−0.01	0.12	No
Psychopathy → ASMR → Anxiety H_5c_	0.10	0.04	2.69	0.00	0.04	0.18	Yes

*β, Beta Value; SE, Standard Error; t, t-statistics; p, significance value; LLCI, lower limit confidence interval; ULCI, Upper Limit Confidence Interval*.

Moreover, ASMR does not help respondents to reduce anxiety and Machiavellianism β = 0.05, t = 1.50, *p* > 0.05, LLCI = −0.01, ULCI = 0.12, there is an insignificant indirect role of ASMR between Machiavellianism and anxiety. Hence H_5b_ is rejected. Finally, ASMR mediates between psychopathy and anxiety β = 0.10, t = 2.69, *p* < 0.05, LLCI = 0.04, ULCI = 0.18, which means that watching ASMR videos help respondents to reduce their anxiety during pandemic times upto 10%. Hence H_5c_ is accepted.

## Discussion

The influence of the dark triad (narcissism, Machiavellianism, and psychopathy) on anxiety disorder during the COVID pandemic was explored. The current study intends to evaluate the mediation function of COVID loneliness and ASMR effects on anxiety disorder in persons with considerably greater dark personality characteristics. This study adds to the body of knowledge about the dark triad, mental health difficulties like anxiety disorder, ASMR impacts on psychological functioning, and loneliness such as social alienation. Thus, the researcher has adopted a cross-sectional research design, while data was collected by using adopted questionnaires from previous studies. For the assumptions testing researcher used partial least square structural equation modeling (PLS-SEM). This software can analyze measurement and structural models simultaneously. In the present research, five study assumptions have been established.

Study assumption 1awas developed to examine the impact of narcissism on anxiety disorder, Statistical results predicted that narcissistic personality disorder was responsible for promoting anxiety and distracting mental health. Findings predicted that vulnerable narcissists typically do not receive the attention and admiration they want to satisfy their strong feeling of entitlement because of their inadequate social competence. The stress they've been exposed to because of this has exacerbated their anxiety in social situations. These findings are in line with (Gogola et al., 2021), and contradict (Akehurst and Thatcher, 2010; Ali and Chamorro-Premuzic, 2010; Lyons et al., 2020) because they found that narcissistic personality characteristics such as excessive self-love, adoration, exhibition, and greater self-esteem might shield people against sadness, anxiety, and depression. However, assumption 1b identified an insignificant association between Machiavellianism and anxiety disorder, and this is due to their diverse social networks and considerable immunization power. These findings contradict those of Sabouri et al. (2016), who reported that Machiavellian persons might suffer from increased anxiety and depression issues as a result of their insincerity and callousness, which can eventually destroy their emotions and senses. Furthermore, study assumption 1c assumed that psychopathy has a positive effect on anxiety disorder while statistics support our assumptions that psychopathic individuals possessed higher anxiety symptoms than those who found higher Machiavellianism. Explaining that egotism, lack of empathy, recidivism, too much self-confidence, unpleasant and abusive talkative attitude, and aberrant sexual acts are all responsible for escalating anxiety and sadness in those with higher psychopathic tendencies. These findings matched those of Derefinko (2015), who claims that psychopaths are connected with higher anxiety and despair than narcissism and Machiavellianism owing to increased impulsivity, low empathy, and coldness. Hence from the above discussion our assumptions 1a and 1c are supported by the data.

The second assumption was that loneliness has a positive effect on anxiety disorder. The findings of the current study are supporting the study assumption by predicting the positive significant effect of loneliness on anxiety disorder. Explaining that social detachment or loneliness is one of the reasons for anxiety disorder. These findings matched with those of others (Teo et al., 2013; Cacioppo et al., 2015), and both studies concluded that loneliness is the root of all mental illnesses. Further, the findings of a longitudinal study conducted by Lim et al. (2016) found that loneliness was the strong predictor of anxiety disorder and mental malfunctioning, underlining the possible importance of loneliness and anxiety disorder on mental health. Rejection from social circles can promote aggression, anxiety, and depression, and this all was experienced during the COVID epidemic (Pedrosa et al., 2020). Furthermore, Cecchetto et al. (2021) found that binge and emotional eating reduced as a result of COVID confinement, indicating that isolation and lockdown have detrimental impacts on emotional health and, consequently, eating behavior. Thus, assumption is supported by the results.

The third assumption has been established to observe the effect of ASMR on anxiety disorder. Our assumption was predicting that AMSR experiences can significantly cope with anxiety disorder. Results have shown that ASMR has a significant negative impact on anxiety disorder. Clarifying that intentionally ASMR tingles, whispers, and sensational stimulus has been related to numerous self-reported benefits, including relaxation, better sleep, reduced stress, and anxiety during the time of survival or depression. These results are in line with Barratt and Davis (2015) wherein in their study majority of respondents claim that ASMR helps them to deal with anxiety disorder. Moreover, these results are also matched with Roberts et al. (2019), who found a weak correlation between anxiety and ASMR and concluded that peoples with anxiety and mental illness mostly prefer whispering, tingling, crisp noises, and slow-motion music because those experiences can help them to control mental stress and anxiety. Hence, from the above discussion our assumption is supported by data.

Additionally, in assumption4awe expected that loneliness has mediated narcissism and anxiety disorder, From the results, it was observed that loneliness has positively and significantly mediated the relationship between narcissism and anxiety disorder. Predicting that social detachment from loved ones can intensify narcissists' anxiety and depression. The ability to empathize appears to be linked to one's level of narcissism. Narcissists' refusal to focus on the needs of others, even when they're not alone, makes it tough to be around them even when they're not alone. Results are in line with Twenge and Campbell (2003). Where, researchers found that due to social rejection or loneliness high narcissists found more aggressive, angry, depressed, and anxious, while in the same study narcissist with more social acceptance reported being relaxed and less anxious. Their results suggested that loneliness was the strong predictor of aggression and anxiety in the high narcissists. Furthermore, in assumption 4b it was found that loneliness has a negative insignificant mediation effect in the relationship between Machiavellianism and anxiety disorder. Meaning that in time of loneliness or social detachment Machiavellian personalities can easily cope with mental health issues because of strong political and proactive behavior in social gatherings. Besides these results, Zhang et al. (2015) found that people with strong Machiavellianism had low emotional intelligence and increased experiences of loneliness. According to Jonason and Krause (2013), Machiavellian personalities might suffer from high levels of loneliness due to high attachment and social contacts. Further, assumption 4c predicted that psychopathy and anxiety disorder is mediated by loneliness. It was observed that loneliness positively mediated between psychopathy and anxiety disorder. Meaning that psychopathic traits including a deceptively appealing appearance, high IQ, pathological egocentricity, and incapacity to love can exacerbate anxiety, feeling of loneliness and psychiatric issues. These findings backed up (Zhang et al., 2015) findings that loneliness is associated with the psychopathic condition and its impact on psychological functioning. Hence from the discussion, statistical results supporting our assumptions 4a and 4c.

In study assumptions5a, 5b, and 5c it was expected that ASMR had mediated between explanatory variables (narcissism, Machiavellianism, and psychopathy) and anxiety disorder respectively. From the statistical results, it was found that ASMR significantly and positively mediated the relationship between narcissism and anxiety disorder, and between psychopathy and anxiety disorder respectively. Results predicting that listening and watching ASMR stuff have reduced narcissists and psychopaths' psychological issues like anxiety, stress, depression and social detachment phobia. That was due to their too much self-love, self-centeredness, and mental toughness. Besides this an insignificant effect was found between Machiavellianism and anxiety disorder. As Gomes Arrulo et al. (2021), found that narcissists can alleviate anxiety conditions by using ASMR items. Narcissism predicted indirect variation in anxiety and performance. In the same study it was observed that narcissists who preferred classical music manifested better stress regulation than those with average and low classical music preference. Additionally, the covid pandemic did not affect Machiavellian anxiety disorder, contrary to Culotta (2013), because Machiavellians have higher loneliness inclination in times of anxiety and depression, and this relationship is stronger than other dark traits (Petrides et al., 2011). Finally, psychopathic individuals claimed ASMR's beneficial effect on anxiety disorder. Anxiety problems may be easily controlled by psychopaths with great willpower, social charm, and ever-green personality traits using ASMR material like music, movies, and social media platforms. Hence study assumptions 5a and 5c were supported by data.

## Conclusion

The COVID epidemic hampered professional and everyday mental health. Anxiety, melancholy, and social estrangement were among the leading causes of mental distractions. Despite personality disorders, the dark triad has also intensified psychological and mental disorders. This inflow has long-term effects on both personal and professional life. However, ASMR helped people with strong interpersonal and psychological strengths relax and reduce anxiety. Like the ‘shivers down the spine' sensation that some people receive while listening to music and intense aesthetic experiences, ASMR appears to have a positive influence on psychiatric conditions, i.e., an anxiety disorder. Anxiety disorder, narcissism, Machiavellianism, and psychopathy were examined through the lenses of attachment theory and cognitive dissonance theory. Loneliness and ASMR research are limited. Moreover, no study examined ASMR and loneliness concerning dark triad and anxiety disorder. Recent studies were conducted on ASMR, loneliness (Zhang et al., 2015), and a study on dark triad and anxiety by Gogola et al. (2021). No study has been undertaken to support the framework described in this article. Another significant feature of this study is that the variables (dark triad, loneliness, ASMR, and anxiety) have never been described in a single study. This study will add to the existing body of knowledge. The current study successfully applied attachment and cognitive dissonance theories. The COVID pandemic impacted everyone globally, but those with dark flux had more anxiety symptoms. The results also showed that narcissists and psychopaths had more anxiety than Machiavellians. Those with higher narcissism and psychopathy report that watching ASMR content throughout the pandemic has a pleasant effect on their mental health and anxiety problems.

The autonomous sensory meridian response is so effective for maintaining mental health. It describes tingling sensations in the head. Individuals watch audio and videos of ASMR such as ear eating, whispering, mouth sounds, fabric scratching, hand movements, tapping, and a lot of other tingles available on YouTube which promote sleep and relaxation among individuals during difficult times, especially pandemics. ASMR has attracted the attention of thousands of individuals during the pandemic. On the other hand, the number of people producing ASMR videos has drastically increased as well. The majority of females are involved in creating ASMR videos for people to watch on YouTube. ASMR also reduces heart rate and increases pleasant effects. It has therapeutic advantages for physical and mental health. Despite having several benefits of ASMR tingles, it has some limitations, such as the effects of ASMR videos might have a low level of effects in a laboratory setting as compared to daily life routine. Second, those respondents having ASMR experiences have experienced a change in physiology, while those who have not experienced ASRM might not have experienced any changes.

### Implications

This study has implications for those who are involved in producing ASMR videos as well as those professionals who are feeling lonely and have high anxiety stress and depression problems. In the dark times of pandemics, professionals can take advantage of ASMR experts and their content produced and can reduce their loneliness and anxiety in dark and tough times. For instance, concerning the cascade of psychological and behavioral effects triggered by the COVID pandemic, it has been shown that the negativity of the psychological effects of the lockdown was further modulated by personality traits (Osimo et al., 2021) and that these effects were also correlated with behavioral wellbeing such as emotional eating (Bowes et al., 2018; Cecchetto et al., 2021). Linking these previous studies to the present one could provide new perspectives on how the present study could further ameliorate COVID-related assessments, e.g. taking into account the possible associations between ASMR and loneliness, personality traits, and behavioral wellbeing.

Loneliness indirectly plays its role in affecting anxiety in narcissist and psychopath individuals, there are complementary mediating effects of loneliness between narcissism psychopathy (Bowes et al., 2018; Cecchetto et al., 2021) and anxiety, but loneliness does not play its indirect role for Machiavellianism (Bowes et al., 2018), thus it is no effect non-mediation as per criteria given by Hair et al. (2017). It suggests that lockdown has detrimental effects on those with dark personalities, increasing anxiety, and depression. Loneliness, on the other hand, has little influence on those with Machiavellian traits. Furthermore, ASMR videos and tingling sensations help narcissists and psychopaths maintain their mental health throughout pandemics and lockdowns whereas Machiavellians do not like to watch and enjoy tingles through ASMR videos.

### Limitations and Future Directions

Instead of several theoretical and practical implications and contributions, the current study has few limitations. Which are essential to reporting and will open new doors for future studies and researchers to contribute toward a body of knowledge. The first limitation is sample size and second limitation is professional sector. One must be careful while generalizing the findings to other sectors. In the future professionals from other sectors such as health, manufacturing, services sector, education, finance, hospitality, tourism, leisure, could be studied. Second, the method used is a single method in future mix methods and longitudinal studies might be helpful to understand the subject matter in depth.

## Data Availability Statement

The datasets generated for this study are available on request to the corresponding author.

## Ethics Statement

Ethical review and approval was not required for the study on human participants in accordance with the local legislation and institutional requirements. Written informed consent from the [patients/participants or patients/participants legal guardian/next of kin] was not required to participate in this study in accordance with the national legislation and the institutional requirements.

## Author Contributions

LS contributed to the data curation. LS, AK, and WW contributed to the formal analysis and original draft. MF, AK, WW, and LS contributed to the revision of the manuscript. YM contributed to the supervision and guidelines. IU and DR contributed to the review and formatting of the manuscript. AK and WW contributed to the conceptualization. KK contributed to the writing-review and editing of the manuscript. All authors contributed to the article and approved the submitted version.

## Funding

This article was supported by National Natural Science Foundation of China (Grant No: 72074039).

## Conflict of Interest

The authors declare that the research was conducted in the absence of any commercial or financial relationships that could be construed as a potential conflict of interest.

## Publisher's Note

All claims expressed in this article are solely those of the authors and do not necessarily represent those of their affiliated organizations, or those of the publisher, the editors and the reviewers. Any product that may be evaluated in this article, or claim that may be made by its manufacturer, is not guaranteed or endorsed by the publisher.
